# Towards a core outcome set for hemorrhoidal disease—a systematic review of outcomes reported in literature

**DOI:** 10.1007/s00384-018-3046-2

**Published:** 2018-04-22

**Authors:** R. R. van Tol, E. van Zwietering, J. Kleijnen, J. Melenhorst, L. P. S. Stassen, C. D. Dirksen, S. O. Breukink

**Affiliations:** 1 Department of Surgery, Maastricht University Medical Centre, P. Debyelaan 25, 6202 AZ Maastricht, The Netherlands; 2 Care and Public Health Research Institute, Department of Family Practice, Maastricht University, Universiteitssingel 40, 6229 ER Maastricht, the Netherlands; 3 Care and Public Health Research Institute, Department of Clinical Epidemiology and Medical Technology Assessment, Maastricht University Medical Centre, P. Debyelaan 25, 6202 AZ Maastricht, The Netherlands; 4 Academic Hospital Maastricht, PO box 5800, 6202 AZ Maastricht, The Netherlands

**Keywords:** Review, Core outcome set, Outcomes, OMERACT, Hemorrhoids

## Abstract

**Purpose:**

Previously published literature regarding treatment of hemorrhoidal disease (HD) revealed a lack of uniform defined outcomes. These differences between outcomes among studies limit transparency and lead to incomparability of results. The aim of this study was to systematically list the types of outcomes used in HD studies. This list will be used to develop a core outcome set.

**Methods:**

We searched Medline (Pubmed), Embase (OVID), and Cochrane for interventional studies for adult patients with HD. Two authors independently identified and reviewed eligible studies. This resulted in a list of outcomes reported by each clinical trial. All outcomes were categorized using the conceptual framework OMERACT filter 2.0.

**Results:**

A total of 34 randomized controlled trials and prospective observational studies were included in this study. A total of 59 different types of outcomes were identified. On average, 5.8 different outcomes (range 2–8) were used per study. The outcomes were structured into three core areas and10 ten domains. The most commonly reported core area was pathophysiological manifestations including the domain symptoms, complications, and recurrence. The most frequently reported outcomes were pain (91%), blood loss (94%), prolapse (71%), and incontinence (56%). There was a high variation in definitions of the common outcomes. And often there was no definition at all.

**Conclusion:**

This study shows a substantial heterogeneity in the types of outcomes in HD studies. We provided an overview of the types of outcomes reported in HD studies and identified a list of potentially relevant outcomes required for the development of a COS.

**Electronic supplementary material:**

The online version of this article (10.1007/s00384-018-3046-2) contains supplementary material, which is available to authorized users.

## Introduction

Hemorrhoidal disease (HD) is the commonest anorectal problem. It affects 5–10% of the population with the highest prevalence in people being 45–65 years of age [1]. HD is usually classified by their location and by the severity of prolapse. The most widely accepted classification is Golighers’ classification [2]. There is considerable variation in the way that HD is managed due to the lack of strong recommendations in treatment guidelines [3, 4]. Basic treatment of HD consists of diet, lifestyle changes, and application of topical ointments [5]. The next treatment modality is often an office-based procedure like rubber band ligation (RBL), sclerotherapy, or radiofrequency therapy [6–9]. In case of persistent symptoms and higher grade of HD, patients are usually treated with surgical solutions (e.g., a Doppler-guided hemorrhoidal artery ligation (DGHAL) [10], with or without Recto-Anal-Repair (RAR) [11–13], stapled hemorrhoidopexy [14, 15], or traditional hemorrhoidectomy [16].

Ideally, a meta-analysis will answer the question what the best current treatment option is for HD. This requires that the same types of outcomes are reported and assessed in the same way. However, previously published literature regarding HD highlighted the lack of uniform outcome definition, measurement, and reporting in research data [6, 17, 18]. In order to overcome this, the European Society of Coloproctology (ESCP) recognized the need to define a core outcome set (COS) for HD. A COS is an agreed standardized set of outcomes that should be assessed and reported in clinical trials for a specific clinical area [19].

Since 1992, the Outcome Measures in Rheumatology (OMERACT) group developed many core outcome sets (COSs) according to a framework and methodology (i.e., the OMERACT Filter) for the identification and validation of core outcome measurement sets for use in clinical trials for any health condition [20]. Also Core Outcome Measures in Effectiveness Trials (COMET) [21] and the International Consortium for Health Outcomes Measurement (ICHOM) [22, 23] initiatives developed a guideline for developing a COS.

We chose to follow the OMERACT working group, since their OMERACT filter resulted in successful development and implementation of core domain and measurement sets for many different diseases [24–30]. In addition, they recently provided the OMERACT Filter 2.0, a practical framework to develop and validate domains and measures for any health condition [31].

The aim of this systematic review was to provide an overview of the types of outcomes reported in studies regarding HD and to identify a list of potentially relevant outcomes for development of a COS.

## Methods

### Literature search

We conducted a search of standard electronic databases such as Medline (Pubmed), Embase (OVID), and Cochrane for relevant studies regarding hemorrhoidal disease. Boolean operators (AND, OR) were used to narrow and widen the search results. In addition, the reference list of the included studies was also searched for additional studies that were not identified in the database searches. Reviews were cross-checked to identify missing studies.

The search was limited to recent published studies between January 2012 and December 2016 representatively for all published studies. No language restrictions were imposed. The full search query is described in Appendix 1.

### Study selection

For inclusion in the review, a study had to meet the following criteria: (1) both randomized controlled trials and (prospective) observational studies since outcomes may have not been reported in RCTs due to selective reporting bias, (2) studies had to assess basic treatment (i.e., diet, lifestyle, and physical therapy), office-based procedures (i.e., rubber band ligation, sclerotherapy, and infrared coagulation) or surgical treatment (i.e., DG-HAL with or without mucopexy, stapled haemorrhoidopexy, and the traditional hemorrhoidectomy) regarding hemorrhoidal disease, and (3) reporting at least one outcome.

### Data extraction

Two authors (RT and EZ) independently screened the titles, abstracts, and full papers regarding the in- and exclusion criteria. Studies without data for retrieval or duplicate publications were excluded. Any disagreement in study selection was resolved by consensus or by discussion with a third reviewer (SB).

The same two authors independently extracted data from the included studies: study name, design, size, intervention(s), outcome, and outcome assessment (e.g., instrument). After completing the data extraction, the two independent reviewers discussed the results, and if discrepancy was present, a consensus was reached.

### Data synthesis

**Fig. 1:**
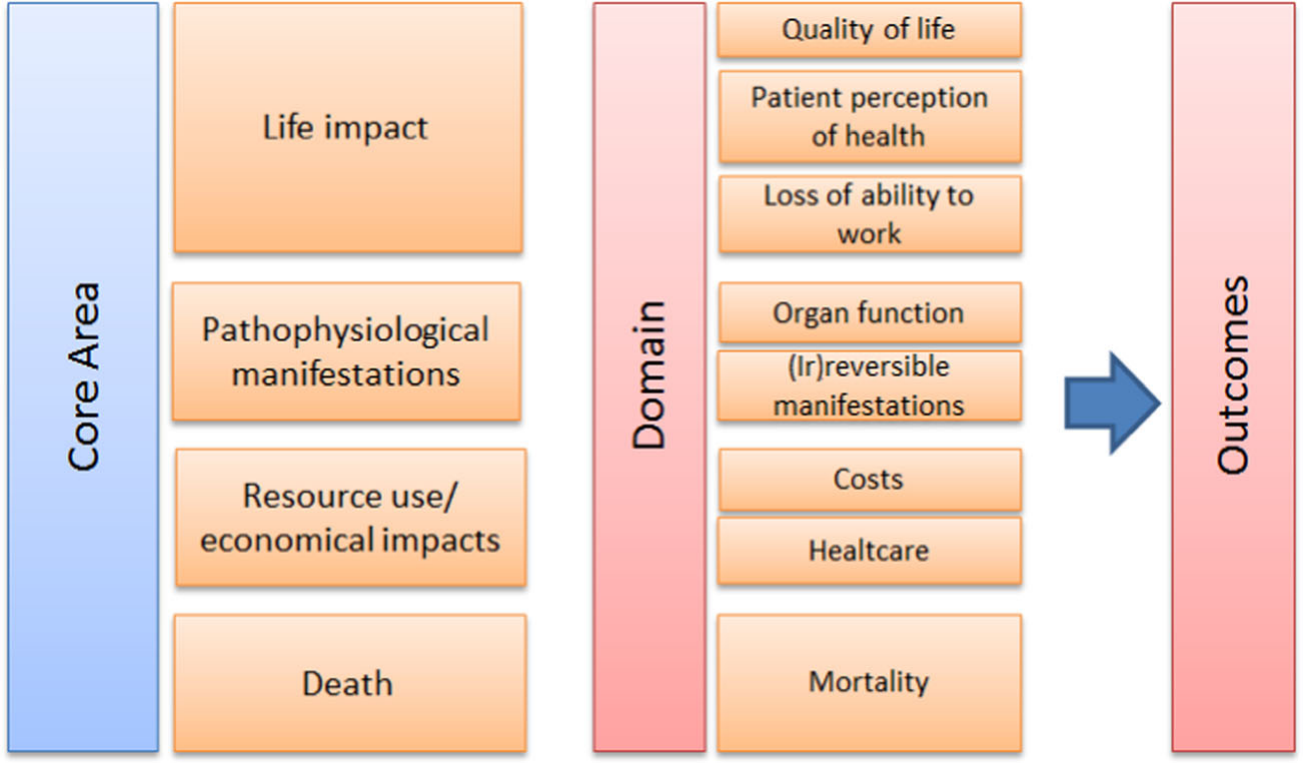
Conceptual framework OMERACT filter 2.0 [32]

Outcomes were categorized according to the framework of the OMERACT 2.0 filter [32, 33]. The framework consisted of four levels including life impact, pathophysiological manifestations, resource use/economic impact, and death. See Fig. 1 for the conceptual framework.

## Results

### Study selection

**Fig. 2:**
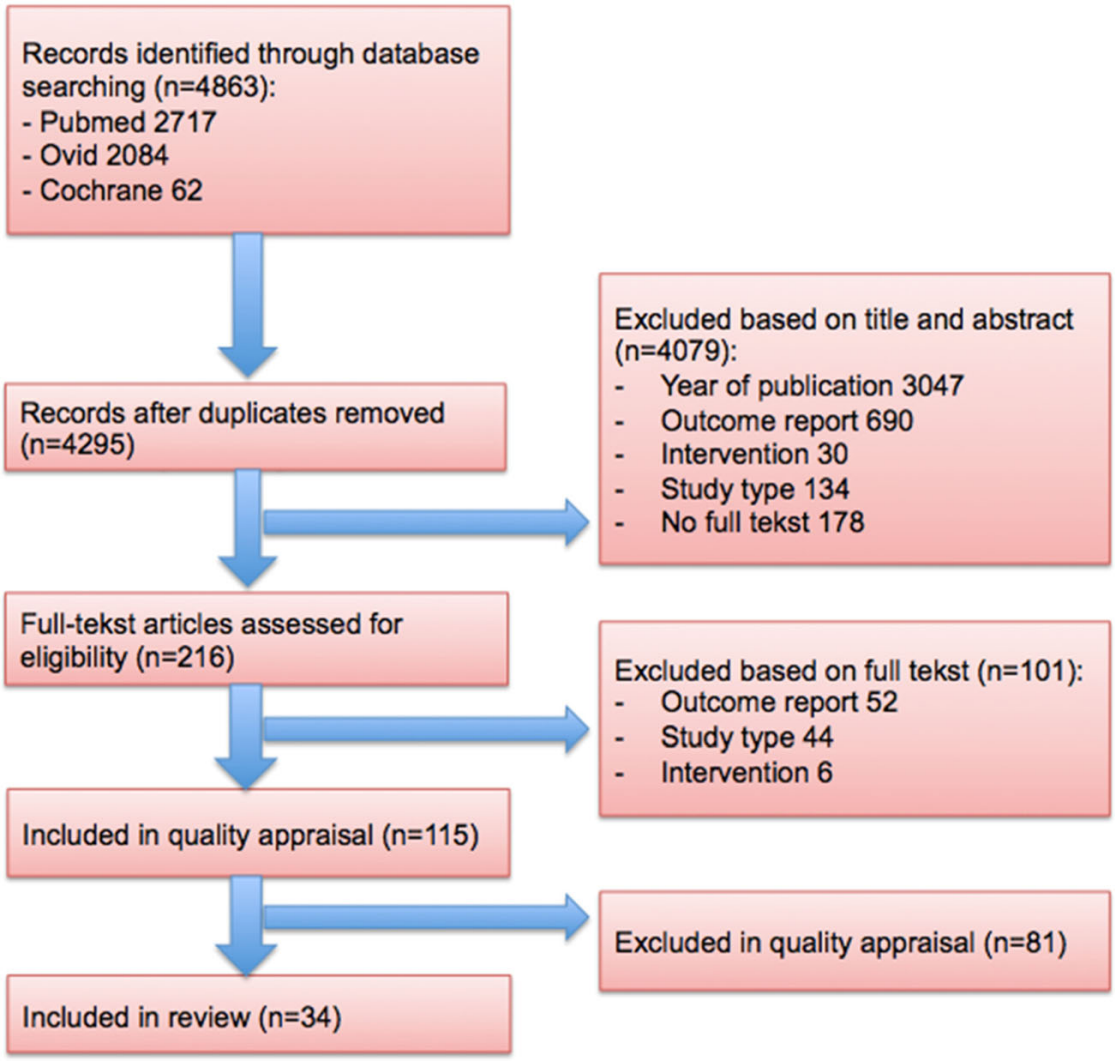
PRISMA flow diagram [34]

The literature search resulted in 4863 abstracts (PubMed, 2717; Ovid, 2084; Cochrane, 62). After deduplication, 4295 records remained and were included in title and abstract screening. Title and abstract screening resulted in the inclusion of 216 articles for full-text screening. After this first step, 34 studies were included in the review. A PRISMA flow diagram of screening and inclusion of studies is shown in Fig. 2. The summary of the characteristics of the included studies according to their year of publication is shown in Appendix 2.

### Study characteristics

Baseline characteristics of the included studies are displayed in Table 1. We identified 24 randomized controlled trials (RCTs) and ten observational studies. Most studies assessed the effectiveness of surgical techniques (92%). In total, 59 different outcomes were used in the 34 included studies. On the average, 5.8 different outcomes [1–6, 32] were assessed per study.

**Table 1:** Baseline characteristics of the included studiesBaseline characteristics of the included studies

Baseline characteristics of the included studies		
		*N* (%)
Total included studies		34 (100)
Study type		
Randomized controlled trials		24 (71)
Observational studies		10 (29)
Treatment	Control	
Surgical treatment		
Hemorrhoidectomy	Hemorrhoidectomy	4 (12)
Hemorrhoidectomy	Stapled hemorrhoidopexy	5 (15)
Hemorrhoidectomy	DG-HAL (with suture mucopexy)	5 (15)
Stapled hemorrhoidopexy	DG-HAL (with suture mucopexy)	3 (9)
DG-HAL (with suture mucopexy)	Suture mucopexy	3 (9)
Hemorrhoidectomy	–	1 (3)
Stapled hemorrhoidopexy	–	1 (3)
DG-HAL (+ RAR or suture mucopexy)	–	6 (18)
Surgical vs office-based procedures		
Hemorrhoidectomy	Sclerotherapy	1 (3)
DG-HAL	Rubber band ligation	1 (3)
DG-HAL	Infrared coagulation	1 (3)
Office-based procedure		
Sclerotherapy	–	1 (3)
Basic treatment		
Diltiazam gel	Placebo	1 (3)
Flavenoids	Placebo	1 (3)
Total different reported outcomes		59
Average number of outcomes per study		5.8 (range 3–8)

### Categorization of outcomes

The 59 different types of outcomes were categorized into three core areas and 10 domains [Appendix 3: Outcomes structured into potential domains and core areas according to OMERACT Filter 2.0]. The core area life impact included the following three domains: patient satisfaction, quality of life, and time to return to normal. The core area pathophysiological manifestations consisted of four domains: symptoms, complications, and recurrence. The core area resource use/economical impact included four domains: duration of operation, duration of hospitalization, re-operation, and costs. No outcome could be placed into the core area death. Apparently, death was not an outcome of interest in hemorrhoidal studies. Therefore, this core area was excluded.

Tables 2, 3, and 4 summarize the number of outcomes reported within each domain and the number of studies reporting these outcomes in each core area.

**Table 2:** Outcome domains in 42 studies included according to OMERACT 2.0 filter core “Life impact”

Domain	Number of outcomes reported within domain	Number of studies reporting outcomes in domain (%)
Patient satisfaction	1	14 (41)
Time to return to normal	6	12 (35)
Quality of life	2	8 (24)

**Table 3:** Outcome domains in 42 studies included according to OMERACT 2.0 filter core “Pathophysiological manifestations”

Domain	Number of outcomes reported within domain	Number of studies reporting outcomes in domain (%)
Symptoms	22	34 (100)
Complications	18	31 (91)
Recurrence	2	20 (59)

**Table 4:** Outcome domains in 42 studies included according to OMERACT 2.0 filter core “Resource use/economical impact”

Domain	Number of outcomes reported within domain	Number of studies reporting outcomes in domain (%)
Duration of operation	3	10 (29)
Duration of hospitalization	2	13 (38)
Re-operation	2	13 (38)
Costs	1	5 (15)

The most reported domains were symptoms (100%), complications (91%), recurrence (59%), and patient satisfaction (41%).

In office-based procedure and basic treatment studies, the most reported domains were similar including symptoms, complications, and patient satisfaction. Recurrence, time to return to normal, quality of life, duration of operation, re-operation, and costs, however, were no outcomes in these studies [Table 5].

**Table 5:** Comparison outcome domains between surgical, office-based, and basic treatment studies according to OMERACT 2.0 filter core.

Domain	Number of surgical studies reporting outcomes in domain (%)	Number of studies comparing surgical vs office-based procedures reporting outcomes in domain (%)	Number of basic treatment and office-based procedure studies reporting outcomes in domain (%)
Patient satisfaction	11 (39)	1 (33)	2 (67)
Time to return to normal	12 (43)	0	0
Quality of life	7 (25)	1 (33)	0
Symptoms	28 (100)	3 (100)	3 (100)
Complications	26 (93)	2 (67)	3 (100)
Recurrence	19 (68)	1 (33)	0
Duration of operation	10 (36)	0	0
Duration of hospitalization	11 (39)	1 (33)	1 (33)
Re-operation	11 (39)	2 (67)	0
Costs	3 (11)	2 (67)	0

### Defining of outcomes

Many outcomes were not clearly defined and assessed using non-validated questionnaires [Appendix 4: describes the type of outcome measurement and the used instruments].

#### Core area life impact

##### Patient satisfaction

Patient satisfaction was used in 14 studies (41%) and all studies used non-validated questionnaires.

##### Time to return to normal

Time to return to normal was used in 12 studies (35%) and was defined as days off to work (*n* = 1), return to work (*n* = 2), resumption of social and working activity (*n* = 1), patients’ time off everyday activity (*n* = 1), time to return to normal activity (*n* = 1), and sick leave (*n* = 1).

##### Quality of life

The outcome quality of life or well-being was reported in eight studies (24%). This domain was assessed by using a Visual Analog Scale (*n* = 1), SF-12 (*n* = 2), or the EQ-5D (QALY) (*n* = 1). In three other studies, assessment was not clear.

#### Core area pathophysiological manifestations

##### Symptoms

Twenty-two different outcomes were used in a “symptom score.” More than one outcome was used in each study (100%). Most reported symptoms were pain (91%), blood loss (94%), and prolapse (71%). Pain was assessed either by a visual analogue scale (*n* = 18), numeric rating scale (NRS) (*n* = 2), brief pain inventory (BPI), (*n* = 2), PATE 2000 (*n* = 1), or not clear (*n* = 8).

Blood loss was assessed using a 4-point scale from “not at all” to “blood dripping in toilet” (*n* = 1), 5-point scale (*n* = 1), visual analog scale (VAS) (*n* = 1), Hemorrhoid symptom score (HSS) (*n* = 1), requiring a re-intervention (transfusion, re-operation) (*n* = 3), or not clear (*n* = 25).

Prolapse was assessed using a 4-point scale: not at all/spontaneously/manual replacement/prolapse remains (*n* = 1), 5-point scale (*n* = 1), PATE 2000 (*n* = 1), or not clear (*n* = 21).

##### Complications

Eighteen different outcomes were used in a “complication score.” In 91% of the studies, more than one outcome was used. The outcome incontinence was the most reported complication and assessed using 5-point scale (*n* = 1), Fecal Incontinence Quality of Life (Rockwood) Scale (FIQOL) (*n* = 2), the Wexner Incontinence Score (*n* = 2), Cleveland Clinic Florida Score (*n* = 2), Fecal Incontinence Quality-of-Life (*n* = 20, the Vaizey Incontinence Score (*n* = 2), or not clear (*n* = 11).

Two studies provided a definition of complications: (1) deviation from normal perioperative course and (2) non-resolving adverse advents related to surgery.

##### Recurrence

Recurrence was used in 20 studies (59%). In nine studies, recurrence was assessed using non-validated questionnaires. One study assessing recurrent prolapse used anoscopic or proctoscopic examination (*n* = 1). In ten studies, the assessment of recurrence was not clear.

However, eleven studies (32%) provided a definition, which varied considerably over studies: “recurrent prolapse and pain” (*n* = 2), “recurrent prolapse” (*n* = 1), “recurrence is based on patient’s complaints and surgeon’s examination” (*n* = 1), “physical examination” (*n* = 1), “relapse symptoms,” “rectal bleeding and impression by the patient of recurrent prolapse” (*n* = 1), “internal hemorrhoids diagnosed on proctoscopy” (*n* = 1), “1 year patient’s self-reported assessment in combination with resource use from their general practitioner and hospital records” (*n* = 1), “clinical evident grade III at 1 year” (*n* = 1), “recurrent prolapse or bleeding or PATE (recurrence of prolapse, acute symptoms, anal tone, and external pill)” (*n* = 1) or “reappearance of the condition at an equal or lower grade than before’ (*n* = 1).

##### Core area resource use/economical impact

Duration of operation was used as outcome in ten studies (29%). Two studies assessed this outcome as incision to application of dressings (*n* = 1) and time between the incision and suturing the skin (*n* = 1).

Duration of hospitalization was used in 13 studies (38%). In all studies, this outcome was not specified.

Re-operation was used in 13 studies (38%). In none of the studies this outcome was specified.

Costs were used in five studies (15%). In none of the studies, this outcome was specified.

## Discussion

This systematic review showed a wide heterogeneity of outcomes in studies reporting on hemorrhoidal disease (HD). Furthermore, a lack of a standardized definition for commonly reported outcomes was observed.

The most commonly reported core area was pathophysiological manifestations including the domain symptoms, complications, and recurrence. All studies reported “symptoms” as an outcome (100%). However, 22 different outcomes were used in this category alone, hence making direct comparison between studies difficult. Most reported symptoms were pain (91%), blood loss (94%), and prolapse (71%). Pain and blood loss were the two pre-eminent symptoms that were addressed by asking the patient. Prolapse was assessed by asking both the patient (51%) and by physical examination by the clinician (49%). This disparity strengthens the case for the development of a COS which embraces the patient voice, as well as those from clinicians. Since patients are fulfilling a more central role in the consulting room, more areas of healthcare intent to develop patient-reported outcomes [35–37]. Until know, there are several non-validated symptom questionnaires developed for HD scoring the frequencies of five outcomes (i.e., pain, blood loss, prolapse, itching, and soiling), for example, the Sodergren score [38].

Besides symptoms, complications were reported in 91% of the studies. This Domain included 18 different outcomes. Again, heterogeneity of outcomes was seen within the studies. Registrations of complications are mandatory since reliable outcome data are crucial to improve outcome of care and gather credible data for benchmarking. In 1992, the Clavien-Dindo classification was introduced offering the possibility to combine grades of complications [39] which enables comparisons to be made between studies. Recurrence was the next frequently reported outcome in the studies (59%) for the core area pathophysiological manifestations. Remarkable, only 11 studies (32%) provided a definition, which varied considerably over studies.

The core area life impact capturing the domain patient satisfaction, time to return to normal, and quality of life was less often used in studies than pathophysiological manifestations. Patient satisfaction was the most reported domain in this core area. This domain was used in 41% of the studies and was always assessed by using non-validated questionnaires. In literature, no validated instrument for assessing patient satisfaction with HD treatment could be found. However, we think this domain is important as we know that dissatisfied patients often have worse clinical outcomes [40–43]. Time to return to normal was reported in 35% of the studies. Several definitions were provided in the studies. Quality of life was assessed in eight studies (24%) and only three studies used a validated questionnaire (i.e., SF-12/36 and EQ-5D).

The core area resource use/economical impact including duration of operation, duration of hospitalization, re-operation, and costs were also often used in studies. Duration of hospitalization was the most reported domain in this core area. Duration of hospitalization was used in 13 studies (38%). In none of the studies, a definition was provided. Duration of operation was used as outcome in ten studies (29%). Re-operation was used in 13 studies (38%), but without providing a definition. Costs were used in five studies (15%).

The OMERACT handbook states that the core area resource use is only recommended for inclusion if stakeholders decide that this core area is mandatory [33].

The core area death was not an outcome of interest in HD studies. Patients with HD are generally healthy patients undergoing elective medical therapy and therefore mortality due to treatment of HD is very rare.

Although HD is the most common anorectal problem, there is no core outcome set (COS) for this condition. This study supports the need to develop a COS for HD to ensure that study results can be compared and combined in the future. Next step will be a consensus study with both patients and healthcare professionals using the Delphi methodology [22]. With this review study, we have derived potentially relevant outcome domains for the development of such COS for HD (47).

Treating HD in daily practice includes often a combination of basic treatment, office-based procedures and surgical treatment, depending on the grade of severity of the disease. Therefore, we included all interventional studies in this review ranging from life styles and diet advices till surgical interventions. We recognize that in doing so, some outcomes (e.g. prolapse) may not be relevant for all types of treatment for HD, especially in low-grade HD. Further, most domains were similar between the studies; however, the domains time to return to normal, quality of life, recurrence, duration of operation, re-operation, and costs were not reported in studies investigating only basic treatment or office-based procedures. This review can serve as a basis for developing a COS for all grades of HD; however, one should be aware of these findings.

The main limitation of our study is that in our search for outcomes, it has been limited to those reported in the existing literature, potentially missing outcomes that are important to other stakeholder groups, in particular patients. The importance of engaging patients in research is being increasingly recognized [44–46]. By asking patients which outcomes should be assessed, we can be confident that treatment interventions are investigated in a way that is relevant to the target population. Therefore, patients will be included in the consensus study. Secondly, the OMERACT Filter may have some limitations. It was developed primarily for trials within the field of rheumatology. Hence, there may be specialty-specific factors that affect the suitability of the filter for other fields. Thirdly, in assigning outcomes to OMERACT core areas, we encountered several examples where an outcome term could potentially be assigned to more than one core area. For example, requirement of repeat procedure could be considered in the context of (ongoing) “Pathophysiological Manifestations,” or within “Resource Use.”

## Conclusion

In Conclusion, this systematic review showed a wide heterogeneity of outcomes in studies reporting on hemorrhoidal disease (HD) and a lack of a standardized definition for commonly reported outcomes. To improve transparency between studies and facilitate the ability to compare and combine (future) studies, we need to develop a core outcome set (COS) for HD. This study, in which we identified a list of potentially relevant outcomes, is the first step towards the development of a COS for HD.

## Electronic supplementary material

ESM 1 (DOCX 55 kb)
