# Supplementary material for: Towards a core outcome set for hemorrhoidal disease—a systematic review of outcomes reported in literature
Source: Int J Colorectal Dis. 2018 Apr 22;33(7):849–56. doi: 10.1007/s00384-018-3046-2 (PMC6002447; doi:10.1007/s00384-018-3046-2)
Supplement: Supplementary file 1 — (DOCX 55 kb) [file 384_2018_3046_MOESM1_ESM.docx]

**Supplementary material**

**Appendix 1**

**Search strategies**

Medline (Pubmed):

| \|  \| \| --- \| \|  \|   #1,"Search (((((haemorrhoid*[Title/Abstract]) OR hemorhoid*[Title/Abstract]) OR haemorhoid*[Title/Abstract]) OR hemorroid*[Title/Abstract]) OR haemorroid*[Title/Abstract]) OR hemoroid*[Title/Abstract]",  #2,"Search hemorrhoid[MeSH Terms]",  #3,"Search (((((((((((surgery[MeSH Subheading]) OR surgery) OR surgical procedures, operative[MeSH Terms]) OR surgical) AND procedures) AND operative) OR operative surgical procedures) OR surgery) OR general surgery[MeSH Terms]) OR general)  #4,"Search ((((treatment[MeSH Subheading]) OR therapy) OR treatment) OR therapeutics[MeSH Terms]) OR therapeutics"  #5,"Search (recurrence[MeSH Terms]) OR recurrence",  #6,"Search ((((diagnosis[MeSH Subheading]) OR diagnosis) OR symptoms) OR diagnosis[MeSH Terms]) OR symptoms",  #7,"Search ((((outcome assessment health care[MeSH Terms]) OR patient outcome assessment[MeSH Terms]) OR treatment outcome[MeSH Terms]) OR clinical effectiveness) OR treatment effectiveness",  (#1 OR #2)  #8,"Search ((hemorrhoid* [Title/Abstract]) OR ((((((haemorrhoid*[Title/Abstract]) OR hemorhoid*[Title/Abstract]) OR haemorhoid*[Title/Abstract]) OR hemorroid*[Title/Abstract]) OR haemorroid*[Title/Abstract]) OR hemoroid*[Title/Abstract])) OR hemorrhoid[MeSH Terms]",  (#2 OR #3)  #9,"Search (((((((((((((surgery[MeSH Subheading]) OR surgery) OR surgical procedures, operative[MeSH Terms]) OR surgical) AND procedures) AND operative) OR operative surgical procedures) OR surgery) OR general surgery[MeSH Terms]) OR general) AND surgery) OR general surgery)) OR (((((treatment[MeSH Subheading]) OR therapy) OR treatment) OR therapeutics[MeSH Terms]) OR therapeutics)",  (#5 OR #6 OR #7)  #10,"Search ((((recurrence[MeSH Terms]) OR recurrence)) OR (((((diagnosis[MeSH Subheading]) OR diagnosis) OR symptoms) OR diagnosis[MeSH Terms]) OR symptoms)) OR (((((outcome assessment health care[MeSH Terms]) OR patient outcome assessment[MeSH Terms]) OR treatment outcome[MeSH Terms]) OR clinical effectiveness) OR treatment effectiveness)"  (#8 AND #9 AND #10)  #11,"Search (((((hemorrhoid* [Title/Abstract]) OR ((((((haemorrhoid*[Title/Abstract]) OR hemorhoid*[Title/Abstract]) OR haemorhoid*[Title/Abstract]) OR hemorroid*[Title/Abstract]) OR haemorroid*[Title/Abstract]) OR hemoroid*[Title/Abstract])) OR hemorrhoid[MeSH Terms])) AND ((((((((((((((surgery[MeSH Subheading]) OR surgery) OR surgical procedures, operative[MeSH Terms]) OR surgical) AND procedures) AND operative) OR operative surgical procedures) OR surgery) OR general surgery[MeSH Terms]) OR general) AND surgery) OR general surgery)) OR (((((treatment[MeSH Subheading]) OR therapy) OR treatment) OR therapeutics[MeSH Terms]) OR therapeutics))) AND (((((recurrence[MeSH Terms]) OR recurrence)) OR (((((diagnosis[MeSH Subheading]) OR diagnosis) OR symptoms) OR diagnosis[MeSH Terms]) OR symptoms)) OR (((((outcome assessment health care[MeSH Terms]) OR patient outcome assessment[MeSH Terms]) OR treatment outcome[MeSH Terms]) OR clinical effectiveness) OR treatment effectiveness))", | 1761,06:11:16  4871,06:12:15  4051959,06:15:19  9498187,06:16:05  406696,06:16:22  8982014,06:17:16  1197546,06:19:38  6602,06:21:03  10554636,06:21:22  9328185,06:21:54  2717,06:22:16 |
| --- | --- | --- | --- |

Cochrane:

1. #1, "hemorrhoid*":ti,ab,kw (Word variations have been searched)
2. #2, haemorrhoid*
3. #3, hemorrhoid*
4. #4, haemorroid*
5. #5, hemorroid*
6. #6, haemorhoid*
7. #7, hemorhoid*
8. #8, haemoroid*
9. #9, hemoroid*
10. #1 or #2 or #3 or #4 or #5 or #6 or #7 or #8 or #9
11. #11, surgery
12. #12, surgical procedures
13. #13, general surgery
14. #14, procedures
15. #15, treatment
16. #16, therapy
17. #17, therapeutics
18. #11 or #12 or #13 or #14 or #15 or #16 or #17
19. #19, recurrence
20. #20, outcome assessment
21. #21, treatment outcome
22. #19 or #20 or #21
23. #10 and #18 and #22

Ovid (Embase):

1. #1, hemorrhoid*.mp. or exp hemorrhoid/

2. #2, haemorrhoid.mp. or exp hemorrhoid/

3. #3, hemorroid.mp.

4. #4, haemorroid.mp.

5. #5, hemoroid.mp.

6. #6, haemorhoid.mp.

7. #7, hemorhoid.mp.

8. #1 or #2 or #3 or #4 or #5 or #6 or #7

9. #9, exp general surgery/ or exp surgery/ or surgery.mp.

10. #10, surgical procedures.mp. or exp surgical technique/

11. #11, treatment.mp.

12. #12, exp therapy/ or therapy.mp.

14. #9 or #10 or #11 or #12

15. #14, recurrence.mp. or exp recurrent disease/

16. #15, exp diagnosis/ or diagnosis.mp.

17. #16, exp symptom assessment/ or exp symptom/ or symptoms.mp.

18. #17, outcome assessment.mp. or exp outcome assessment/

19. #18, treatment outcome.mp. or exp treatment outcome/

20. #19, exp treatment outcome/ or exp outcome assessment/ or outcome.mp.

21. 14 or 15 or 16 or 17 or 18 or 19

22. #8 and #14

23. #21 and #22

# Appendix 2

**Included studies**

| **First Author** | **Year** | **Title** | **Study type** | **Treatment** | **Control** | **Primary outcome** | **Other outcomes** |
| --- | --- | --- | --- | --- | --- | --- | --- |
| N. Arslani | 2012 | A Randomized Clinical Trial comparing Ligasure versus Stapled Hemorrhoidectomy. | Randomized Controlled Trial | Ligasure | Stapled Hemorrhoidectomy | No mention | Recurrence, postoperative complications (i.e. urinary retention, early and late bleeding, fecal incontinence and anal stenosis), pain with VAS and analgesic use, wound healing time and patients’ time off everyday activity |
| JP. Schuurman | 2012 | Hemorrhoidal artery ligation procedure with of without Doppler transducer in grade II and III hemorrhoidal disease: a blinded randomized trial. | Randomized Controlled Trial | DG-HAL | HAL | Improvement of self-reported clinical parameters after both 6 weeks and 6 months. | Improvement symptoms (blood loss, pain, prolapse, and problems with defecation, amount of discomfort in daily life), complications, patient's satisfaction rate, postoperative pain (VAS), time for regaining daily activities, need for subsequent treatment |
| C. Ammatoro | 2012 | Stapled haemorrhoidopexy vs Milligan-Morgan haemorrhoidectomy for grade III haemorrhoids: a randomized controlled trial. | Randomized Controlled Trial | Stapled hemorrhoidopexy | Milligan-Morgan hemorrhoidectomy | No mention | Pain (VAS and analgesic use), symptoms (i.e. bleeding, prolapse, anal pain urgency, tenesmus, flatus incontinence), complications (i.e. haemorrhage, urinary retention, thrombosis and sepsis), recurrence (of prolapse), length of hospital stay, return to work, re-operation, patient satisfaction |
| A. Infantino | 2012 | Prospective randomised multicentre study comparing stapler haemorrhoidectomy with Doppler-guided transanal haemorrhoid dearterialization for third degree haemorrhoids. | Randomized Controlled Trial | Stapled Haemorrhoidectomy | DG-HAL | No mention | Pain, early and late complications, bleeding, recurrence of symptoms (including prolapse, and the persistence of other anal symptoms using PATE2000), quality of life, in hospital stay, further treatment |
| N. Zampieri | 2012 | Long-term results and quality of life in patients treated with hemorrhoidectomy using two different techniques: Ligasure versus transanal hemorrhoidal dearterilisation. | Randomized Controlled Trial | Ligasure | Transanal Hemorrhoidal Dearterialisation | Quality of life, quality of defecation, and regression of symptoms | Quality of life, quality of defecation, regression of symptoms, length of surgery, comfort for patients, request analgesics, relapses |
| JS. Kim | 2013 | Stapled hemorrhoidopexy versus Milligan-Morgan hemorrhoidectomy in circumferential third-degree hemorrhoids: long-term results of a randomized controlled trial. | Randomized Controlled Trial | Stapled hemorrhoidopexy | Milligan-Morgan hemorrhoidectomy | Postoperative pain | Long-term recurrence rate, complications (urinary retention and incontinence symptoms), symptoms (bleeding, itching/burning, prolonged wound healing), operative time |
| T. Yano | 2013 | Prospective study comparing the new sclerotherapy and hemorrhoidectomy in terms of therapeutic outcomes at 4 years after treatment. | Observational study | Sclerotherapy | Hemorrhoidectomy | No mention | Symptom free-rate, prolaps, bleeding, satisfaction rates |
| CW. Chen | 2013 | Results of 666 consecutive patients treated withLligasure for symptomatic prolapsed hemorrhoids with a minimum follow-up of 2 years. | Observational study | Hemorrhoidectomy (Ligasure) | No control | No mention | Recurrence, complications (i.e. bleeding, incontinence), operative time, hospital stay, re-operation, anal pain |
| SE. Elmér | 2013 | A Randomized trial of transanal hemorrhoidal deartilization with anopexy compared with open hemorrhoidectomy in the treatment of hemorrhoids. | Randomized Controlled Trial | DG-HAL with mucopexy | Hemorrhoidectomy | Postoperative pain | Complications, improvement of symptoms (anal pain, defecatory bleeding, anal pruritis, soiling and replacement of prolapse), well-being, re-operation, time to return to work |
| PI. Denoya | 2013 | Dearterialization with mucopexy versus haemorrhoidectomy for grade III and IV haemorrhoids: short term results of a double-blind randomized controlled trial. | Randomized Controlled Trial | DG-HAL with mucopexy | Hemorrhoidectomy | Pain | Adverse events defined as the deviation from normal peri-operative course (blood loss, urinary retention and constipation), quality of life (disablility), fecal incontinence, operating time |
| H. Bulus | 2013 | Evalution of two hemorrhoidectomy techniques: harmonic scalpel and Ferguson's with electrocautery. | Randomized Controlled Trial | Hemorrhoidectomy (Harmonic scalpel) | Ferguson hemorrhoidectomy | No mention | Operating time, postoperative pain, length of hospital stay, time to return to normal activity, postoperative complications (abscess, stenosis, incontinence), recurrence |
| A. Ahmad | 2013 | Comparative analysis of Doppler Guided hemorrhoidal artery ligation (DG-HAL) & infrared Coagulation (IRC) in Management of Hemorrhoids. | Randomized Controlled Trial | DG-HAL | Infrared Coagulation | No mention | Time taken in the procedure, perioperative pain, cost of treatment, duration of hospital stay, symptomatic relief, postoperative complications, requirement of repeat procedure |
| R. Yamoul | 2013 | The effectiveness of Doppler controlled hemorrhoidal artery ligation based on preliminaries results. | Observational study | DG-HAL | No control | No mention | Duration of surgery, duration of hospitalization, complications, postoperative pain, satisfaction, symptoms (pain, bleeding, prolapse and discomfort), re-operation |
| T. Sugimoto | 2013 | A randomized, prospective, double-blind placebo-controlled trials of the effect of ditiazem gel on pain after hemorrhoidectomy | Randomized Controlled Trial | Diltiazem gel | Placebo | No mention | Pain, operative time, bloodloss, complications (itching sensation, headache, and dizziness), length of hospital stay, patient satisfaction |
| P. Denoya | 2014 | Hemorrhoidal dearterialization with mucopexy versus hemorrhoidectomy: 3-year follow-up assessment of a randomized controlled trial. | Randomized Controlled Trial | DG-HAL with mucopexy | Hemorrhoidectomy | Recurrence of internal hemorrhoids | Chronic complications (anal stenosis, unhealed wounds, anal fissures and incontinence), symptoms (pain), overall quality of life, re-operation |
| MA. Qarabaki | 2014 | Circular vs three quadrant hemorrhoidectomy for end stage hemorrhoids: short-and-long-term outcomes of a prospective randomized trial. | Randomized Controlled Trial | Circular hemorrhoidectomy | Three quadrant hemorrhoidectomy | No mention | Complications (urinary retention, hemorrhage, wound infection, fever, inflammatory reaction, anal stricture), prolapse recurrence, operative time, length of hospital stay, time to return to normal activity, overall satisfaction, symptoms (anal pain, discomfort, bleeding, mass swelling and itching) |
| P. De Nardi | 2014 | A prospective randomized controlled trial comparing the short-and long-term results of doppler-guided transanal hemorrhoid dearterialization with mucopexy versus excision hemorrhoidectomy for grade III hemorrhoids. | Randomized Controlled Trial | DG-HAL witch mucopexy | Hemorrhoidectomy | Postoperative pain | Symptoms (bleeding discomfort, pain, prolapse), postoperative complications (incontinence), resumption of social and working activity, patient satisfaction, re-intervention |
| A. Talha | 2014 | Ligasure, Harmonic Scalpel versus conventional diathermy in excisional haemorrhoidectomy: a randomized controlled trial. | Randomized Controlled Trial | Haemorrhoidectomy (Ligasure) | Conventional diathermy in excisional haemorrhoidectomy | No mention | Operative time, symptoms, postoperative pain, complications (haemorrhage and urine retention), wound healing rate |
| V. Ripetti | 2015 | A Randomized Trial Comparing Stapled Rectal Mucosectomy Versus Open and Semiclosed Hemorrhoidectomy. | Randomized Controlled Trial | Stapled hemorrhoidopexy | Hemorrhoidectomy (Open and Semiclosed) | Postoperative pain | Pre-operative symptoms (pain, soiling, bleeding, ichting), return to work activity, duration of surgery, days required for return to physical activity, days required for complete healing (the disappearance of all of the symptoms linked to the surgical procedure), recurrence, complications |
| C. Ratto | 2015 | Doppler-guided transanal haemorrhoidal dearterialization for haemorrhoids: results from a multicentre trial. | Observational study | DG-HAL met mucopexy | No control | No mention | Complications (urinary retention, thrombosis, constipation, anal abscess/infection, fissure), recurrence, symptoms (bleeding, prolapse, manual reduction, impact of quality of life and discomfort/pain), re-operation |
| F. Noguerales | 2015 | Treatment of haemorrhoids by transanal haemorrhoidal dearterialization. Experience of several specialized units. | Observational study | DG-HAL (+mucopexy) | No control | No mention | Postoperative pain, duration of hospital stay, symptoms (pruritis, pain and bleeding), days off from work, recurrence, complications, re-intervention |
| Y. Bilgin | 2015 | Short- and long-term results of harmonic scalpel hemorrhoidectomy versus stapler hemorrhoidopexy in treatment of hemorrhoidal disease. | Randomized Controlled Trial | Harmonic scalpel hemorrhoidectomy | Stapled hemorrhoidopexy | No mention | Presenting symptoms (pain, mucous discharge, pruritus ani, prolapse and flatulence), operative time, duration of hospitalization, return to daily acitivities, postoperative complications, postoperative pain, recurrence |
| I. Giannini | 2015 | Flavenoids mixture (diosmin, troxerutin, hesperidin) in the treatment of acute hemorrhoidal disease: a prospective randomized, triple-blind, controlled trial. | Randomized Controlled Trial | Flavenoids mixture | Placebo | No mention | Symptoms (pain, bleeding and itching), stool (Bristol stool scale), complications (edema, prolapse and trombosis) |
| SR. Brown | 2016 | Haemorrhoidal artery ligation versus rubber band ligation for the management of symptomatic second-degree and third-degree haemorrhoids (HubBLe): a multicentre, open-label, randomised controlled trial. | Randomized Controlled Trial | DG-HAL | Rubber band ligation | Recurrent haemorrhoids at 12 months after procedure | Recurrence, postoperative pain, complications, persistent symptoms (HSS), need for further treatment, costs, quality of life |
| F. Aigner | 2016 | Doppler-guided haemorrhoidal artery ligation with suture mucopexy compared with suture mucopexy alone for the treatment of Grade III haemorrhoids: a prospective randomized controlled trial. | Randomized Controlled Trial | DG-HAL with suture mucopexy | Suture mucopexy alone | Postoperative pain | Recurrence, anatomical (residual prolapse, alerations of vascularization of anorectal vascular plexus), haemorrhoidal symptoms (bleeding, urgency, discharge, pruritus) |
| C. Hoyuela | 2016 | HAL-RAR (Doppler guided haemorrhoid artery ligation with recto-anal repair) is a safe and effective procedure for haemorrhoids. Results of a prospective study after two-years follow-up. | Observational study | DG-HAL with Recto-anal-Repair (RAR) | No control | No mention | Postoperative pain, complications (incontinence, urgency, soiling), symptoms (bleeding, prolapse, itching, pain and soiling), satisfaction, recurrence, operating time, re-operation |
| DR. Lim | 2016 | Comparison of a Hemorrhoidectomy With Ultrasonic Scalpel Versus a Conventional Hemorrhoidectomy. | Randomized Controlled Trial | Hemorrhoidectomy with Ultrasonic scalpel | Conventional hemorrhoidectomy | No mention | Postoperative pain, complications (bleeding, urinary retention, abcess, gangrene,stricture, incontinence), operation time, duration hospital stay |
| S. Leardi | 2016 | Doppler-Guided Transanal Hemorrhoidal Dearterialization (DG-THD) Versus Stapled Hemorrhoidopexy (SH) in the Treatment of Third-Degree Hemorrhoids: Clinical Results at Short and Long-Term Follow-Up. | Randomized Controlled Trial | Doppler-Guided Transanal Hemorrhoidal Dearterialization | Stapled hemorrhoidopexy | No mention | Postoperative pain, symptoms (pain, bleeding, first bowel movement), return to daily activities, recurrence, re-operation, postoperative complications, satisfaction |
| A. Voigtsberger | 2016 | Stapled hemorrhoidopexy: functional results, recurrence rate, and prognostic factors in a single center analysis. | Observational study | Stapled hemorrhoidopexy | No control | No mention | Complications (bleeding, incontinence), functional dysorders (anal stenosis and irritation), recurrence |
| A. Zakharchenko | 2016 | Safety and efficacy of superior rectal artery embolization with particles and metallic coils for the treatment of hemorrhoids (Emborrhoid technique). | Observational study | Superior rectal artery embolization with particles and metallic coils | No control | No mention | Complications (hematomas, infections or psuedoaneurysms), symptoms (irritation, discomfort, bloody discharge and pain), patient satisfaction, anatomical (sphincter tone) |
| HX. Huang | 2016 | Application of 'tying, binding and fixing operation' in surgical treatment of severe mixed hemorrhoids. | Observational study | Tying, binding and fixing operation | No control | No mention | Symptoms (hemorrhage, prolapse improvement, shrinkage hemorrhoid volume, preservation of anal function), satisfaction, lengt of hospital stay, costs, recurrence rate, complications (hemorrhage, infection, thrombosis, anal enema and urination), operating time |
| M. Zhai | 2016 | A Randomized Controlled Trial Comparing Suture-Fixation Mucopexy and Doppler-Guided Hemorrhoidal Artery Ligation in Patients with Grade III Hemorrhoids. | Randomized Controlled Trial | DG-HAL vs mucopexy | Suture mucopexy alone | No mention | Clinical symptoms, postoperative recurrence, complications (bleeding, anal discomfort, urinary retention, anal stricture and fecal incontinence), duration of hospilization, costs of the procedure, patients' satisfaction |
| M. Basile | 2016 | Transanal anopexy with HemorPex System (HPS) is effective in treating grade II and III hemorrhoids: medium-term follow-up. | Observational study | Transanal anopexy with HemorPex System (HPS) | No control | No mention | Complications (pain, bleeding thrombosis), recurrence, patient satifaction, symptoms (pain, bleeding, prolapse and difficulties with hygiene) |
| PA. Lehur | 2016 | Cost-effectiveness of New Surgical Treatments for Hemorrhoidal Disease: A Multicentre Randomized Controlled Trial Comparing Transanal Doppler-guided Hemorrhoidal Artery Ligation With Mucopexy and Circular Stapled Hemorrhoidopexy. | Randomized Controlled Trial | Transanal Doppler-guided Hemorrhoidal Artery Ligation With Mucopexy | Stapled hemorrhoidopexy | Morbidity rate | Adverse events (urinary retention, thombosis, fissue, local infection, incontinence, septic complications, severe bleeding), pain levels, hospital stay, sick leave, cost-effectiveness |

**Appendix 3**

**Outcomes structured into potential domains and core areas according to OMERACT Filter 2.0 (**[**35**](#_ENREF_35)**)**

| **Outcomes** | **Domains** | **Core areas** |
| --- | --- | --- |
| (n=59) | (n=10) | (n=3) |
| Patient satisfaction | Satisfaction | Life impacts |
| Quality of life | Quality of life |  |
| Well-being |  |  |
| Days off to work | Time to return to 'normal' |  |
| Return to work |  |  |
| Resumption social and working activity |  |  |
| Patients’ time off everyday activity |  |  |
| Time to return to normal activity |  |  |
| Sick leave |  |  |
| Prolapse | Symptoms | Pathophysiological manifestations |
| Pain |  |  |
| Bleeding/ blood loss |  |  |
| Itching |  |  |
| Pruritus |  |  |
| Soiling |  |  |
| Discomfort |  |  |
| Urgency |  |  |
| Mucous discharge |  |  |
| Tenesmus |  |  |
| Burning |  |  |
| Mass swelling |  |  |
| Constipation |  |  |
| Hygiene problems |  |  |
| Quality of defecation |  |  |
| Passage of gas/feces |  |  |
| Flatus incontinence |  |  |
| Wound healing |  |  |
| Irritation |  |  |
| Anal function |  |  |
| Anatomical difference |  |  |
| Anal tone |  |  |
| Fecal incontinence | Complications |  |
| Outlet obstruction |  |  |
| External pil |  |  |
| Severe pain |  |  |
| Severe bleeding |  |  |
| Gangrene |  |  |
| Edema |  |  |
| Sepsis |  |  |
| Local infection |  |  |
| Haemorrhage |  |  |
| Hematomas |  |  |
| Thrombosis |  |  |
| Anal fissure |  |  |
| Abscess |  |  |
| Urinary retention |  |  |
| Anal stricture |  |  |
| Unhealed wounds |  |  |
| Anal stenosis |  |  |
| Recurrent prolapse | Recurrence |  |
| Recurrent haemorrhoidal symptoms |  |  |
| Time taken in the procedure | Duration of operation | Resource use/Economical impact |
| Duration of surgery |  |  |
| Operative time |  |  |
| Hospital stay | Duration of hospitalization |  |
| Duration or length of hospitalization |  |  |
| Cost of treatment | Costs |  |
| Requirement of repeat procedure | Re-operation |  |
| (Surgical) re-intervention |  |  |

**Appendix 4**

**Measurement of outcomes and measurement tools used**

| **Outcomes** | **Measurement type used (N)** | | | | **Measurement tools used** |
| --- | --- | --- | --- | --- | --- |
|  | **Patient reported** | **Clinician reported** | **Technical measure** | **Not clear** |  |
| **Life impact** | | | | | |
| Patient satisfaction | 14 | 0 | 0 | 0 | Questionnaire nd (n=14) |
| Quality of life | 7 | 0 | 0 | 0 | Visual Analog Scale (n=1), SF-12 (n=2), EQ-5D (n=1), not clear (n=3) |
| Well-being | 1 | 0 | 0 |  | Questionnaire nd (n=1) |
| Days off to work | 0 | 0 | 0 | 1 | Not specified (n=1) |
| Return to work | 0 | 0 | 0 | 2 | Not specified (n=2) |
| Resumption social and working activity | 0 | 0 | 0 | 1 | Not specified (n=2) |
| Patients’ time off everyday activity | 0 | 0 | 0 | 3 | Not specified (n=3) |
| Time to return to normal activity | 0 | 0 | 0 | 4 | Not specified nd (n=4) |
| Sick leave | 0 | 0 | 0 | 1 | Not specified (n=1) |
| **Pathophysiological manifestations** | | | | | |
| Prolapse | 12 | 11 | 0 | 1 | 4-point scale (n=1), 5-point scale (n=1), PATE 2000 (n=1), not clear (n=21) |
| Pain | 31 | 0 | 0 | 0 | Visual Analog Scale (VAS) (n=18), NRS (n=2), BPI (n=2), PATE2000 (n=1), not clear (n=8) |
|  |  |  |  |  |  |
|  |  |  |  |  |  |
| Bleeding/ blood loss | 29 | 3 | 0 | 0 | 4-point scale from ‘not at all’ to ‘blood dripping in toilet’ (n=1), 5-point scale (n=1), VAS (n=1), Haemorrhoid Symptom Score (HSS) (n=1), requiring a re-intervention (n=3), not clear (n=25) |
|  |  |  |  |  |  |
| Itching | 6 | 0 | 0 | 0 | Not clear (n=6) |
| Pruritis | 4 | 0 | 0 | 0 | Not clear (n=4) |
| Soiling | 8 | 0 | 0 | 0 | Not clear (n=8) |
| Discomfort | 8 | 0 | 0 | 0 | Not clear (n=8) |
| Urgency | 3 | 0 | 0 | 0 | Not clear (n=3) |
| (Mucous) discharge | 3 | 0 | 0 | 0 | Not clear (n=3) |
| Tenesmus | 1 | 0 | 0 | 0 | Not clear (n=1) |
| Burning | 1 | 0 | 0 | 0 | Not clear (n=1) |
| Mass swelling | 1 | 0 | 0 | 1 | Not clear (n=1) |
| Constipation | 1 | 0 | 0 | 0 | Not clear (n=1) |
| Hygiene problems | 1 | 0 | 0 | 0 | Not clear (n=1) |
| Quality of defecation | 1 | 0 | 0 | 0 | Bristol stool scale (n=1) |
| Passage of gas and feces | 1 | 0 | 0 | 0 | Not clear (n=1) |
| Flatus incontinence | 1 | 0 | 0 |  | Not clear (n=1) |
| Wound healing | 1 | 1 | 0 | 1 | Time in weeks needed for complete epithelization (n=1), not clear (n=2) |
| Irritation | 1 | 0 | 0 | 0 | Not clear (n=1) |
| Anal function | 0 | 0 | 0 | 1 | Not clear (n=1) |
| Anatomical difference | 0 | 0 | 1 | 0 | Not clear (n=1) |
| Anal tone | 0 | 0 | 1 | 0 | Sphincter tone (n=1) |
| Fecal incontinence | 19 | 0 | 0 | 0 | 5-point scale (n=1), Cleveland incontinence score (n=2), Fecal Incontinence Quality-of-Life (n=2), Vaizey (n=2), Wexner incontinence score (n=2), not clear (n=11) |
| Outlet obstruction | 0 | 0 | 0 | 1 | Not clear (n=1) |
| External pil | 0 | 0 | 0 | 0 | Not clear (n=1) |
| Gangrene | 0 | 0 | 0 | 1 | Not clear (n=1) |
| Edema | 0 | 2 | 0 | 0 | Not clear (n=2) |
| Sepsis | 0 | 1 | 0 | 0 | Not clear (n=2) |
| Local infection | 0 | 1 | 0 | 0 | Not clear (n=1) |
| Hemorrhage | 0 | 1 | 0 | 0 | Not clear (n=1) |
| Hematomas | 0 | 1 | 0 | 0 | Not clear (n=1) |
| Thrombosis | 0 | 5 | 0 | 0 | Not clear (n=5) |
| Anal fissure | 0 | 1 | 0 | 0 | Not clear (n=1) |
| Abscess | 0 | 2 | 0 | 0 | Not clear (n=2) |
| Urinary retention | 0 | 9 | 0 | 0 | Not clear (n=9) |
| Anal stricture | 0 | 3 | 0 | 0 | Not clear (n=3) |
| Unhealed wounds | 0 | 0 | 0 | 5 | Not clear (n=5) |
| Anal stenosis | 0 | 4 | 0 | 0 | Not clear (n=4) |
| Recurrent prolapse | 1 | 6 | 0 | 0 | Anoscopic or proctoscopic examination (n=1), not clear (n=6) |
| Recurrent haemorrhoidal symptoms | 8 | 1 | 0 | 4 | Questionnaire nd (n=9), not clear (n=4) |
| **Resource use/ economical impact** | | | | | |
| Time taken in the procedure | 0 | 0 | 2 | 0 | From incision to application of dressings (n=1), time between the incision and suturing the skin (n=1) |
| Duration of surgery | 0 | 0 | 2 | 0 | Not specified (n=2) |
| Operative time | 0 | 0 | 6 | 0 | Not specified (n=6) |
| Hospital stay | 0 | 0 | 10 | 0 | Not specified (n=10) |
| Duration or length of hospitalization | 0 | 0 | 3 | 0 | Not specified (n=11) |
| Cost of treatment | 0 | 0 | 4 | 0 | Not specified (n=4) |
| Requirement of repeat procedure | 0 | 8 | 0 | 0 | Not specified (n=8) |
| Surgical reintervention | 0 | 6 | 0 | 0 | Not specified (n=6) |
